# Sustainable Carboxymethyl Cellulose-Based Foams via Deep Eutectic Solvent Processing for pH-Responsive Drug Delivery

**DOI:** 10.3390/jfb17070337

**Published:** 2026-07-12

**Authors:** Bruno B. Ravanello, Filipe Silva de Matos, Bruna Ramos Navalhas, Luís Pereira, Nalin Seixas

**Affiliations:** 1 AlmaScience Colab, Madan Parque, Rua dos Inventores, 2825-182 Caparica, Portugal; bruno.ravanello@almascience.pt (B.B.R.); filipe.matos@almascience.pt (F.S.d.M.); bruna.navalhas@almascience.pt (B.R.N.); lmnp@fct.unl.pt (L.P.); 2 CENIMAT|i3N, Department of Materials Science, School of Science and Technology, NOVA University Lisbon and CEMOP/UNINOVA, Campus da Caparica, 2829-516 Caparica, Portugal; 3 CICECO—Aveiro Institute of Materials, Department of Chemistry, University of Aveiro, 3810-193 Aveiro, Portugal

**Keywords:** deep eutectic solvent, carboxymethyl cellulose, foams, resveratrol, drug delivery

## Abstract

Carboxymethyl cellulose (CMC)-based materials are widely studied for functional materials and porous platform applications, yet their stability usually requires energy-intensive thermal curing or toxic chemical crosslinkers, which limit process sustainability. In this work, we present a more sustainable approach for the preparation of CMC-based foams using deep eutectic solvents (DES) as multifunctional structuring agents. CMC hydrogels were prepared with different DES at room temperature, followed by freeze-drying to obtain foams. Among the tested DES, choline chloride:oxalic acid (1:1) combined with glycerol produced foams with the most favorable properties, including high water uptake (288.24 ± 3.02% after 1 h) and water stability for 28 days. Morphological analysis revealed a homogeneous and interconnected porous network (32.2 ± 13.3 µm), while compression tests demonstrated good mechanical recovery (93.29 ± 3.12% over 10 cycles). Fourier transform infrared spectroscopy suggests interactions between CMC and DES, especially hydrogen bonds. The foams exhibited pH-dependent behavior, with limited resveratrol release under acidic conditions (22.2 ± 4.0% after 24 h), with significant release at pH 7.4 (85.30 ± 5.75%) and total release at pH 13.0. Drug release kinetics suggest a diffusion-controlled mechanism under acidic pH, transitioning to anomalous transport at higher pH values. This study demonstrates that DES can be used to prepare CMC-based foams, providing a more sustainable route to porous materials. Although biological validation is needed to confirm therapeutic safety, this study provides an initial physicochemical basis for using these matrices as tunable and stimuli-responsive porous materials.

## 1. Introduction

Polysaccharide derivatives, such as carboxymethyl cellulose (CMC), are promising candidates for the development of sustainable materials for a wide range of applications due to their chemical versatility, biodegradability, and abundance [1,2,3,4]. As a prominent anionic cellulose derivative, CMC exhibits excellent water solubility and chemical reactivity resulting from the introduction of carboxymethyl groups along the polymer backbone. The abundant hydroxyl and carboxyl functional groups serve as active sites for intermolecular interactions and crosslinking reactions, enabling the formation of several types of materials, such as hydrogels [1,2].

Hydrogels are three-dimensional hydrophilic polymer networks that exhibit a high capacity for water absorption and retention while preserving their structural integrity [3,5]. Their high water content, tunable viscoelasticity, and ability to mimic the extracellular matrix have led to widespread use in several applications, particularly as drug delivery systems [5,6,7,8,9]. Importantly, hydrogels can undergo controlled dehydration processes, such as freeze-drying, leading to the formation of lightweight and open-celled structures called foams, which retain the chemical functionality of the initial hydrogel while offering enhanced porosity and surface area, which are important characteristics for drug release applications [10,11]. The properties of these foams, including swelling, mechanical behavior, and stimuli-responsiveness, are fundamentally governed by the stability and chemical interactions within the hydrogel network [7,12,13].

Traditionally, stabilizing these networks requires the establishment of crosslinks between polymer chains in the initial hydrogel, which can be achieved either by physical interactions, such as hydrogen bonds or ionic complexation, or chemical reactions that introduce covalent bonds [5,7,14]. Common strategies include the use of covalent crosslinkers, like glutaraldehyde, or ionic agents, such as calcium chloride (CaCl_2_) [14,15,16]. Although these strategies are effective, many of them require energy-intensive high-temperature curing or the use of toxic crosslinkers, which can limit the sustainability and process simplicity of the final material [15,16]. For instance, creating CMC-based foams that remain stable in aqueous environments typically requires chemical modifications or reinforcing additives [4,17,18]. Consequently, there is a growing demand for fabrication routes that can produce polysaccharide-based foams with high mechanical robustness and long-term water stability without compromising green chemistry principles [9,14].

In this context, deep eutectic solvents (DES) have emerged as green media for polysaccharide processing. Formed by combining a hydrogen bond donor (HBD) and a hydrogen bond acceptor (HBA), DES can act as a multifunctional structuring agent [19,20,21]. When incorporated into polysaccharide-based systems, DES can interact strongly with polymer chains through hydrogen bonding, influencing polymer chain mobility, water affinity, and network organization [19,21,22,23]. For instance, DES-modified polysaccharide systems have been reported to exhibit enhanced swelling capacity, altered porosity, and improved flexibility, although these effects may also influence mechanical resistance, depending on formulation composition [23,24,25,26]. Despite significant progress, most reported DES-based polysaccharide systems still rely on additional chemical crosslinkers or reinforcing agents to achieve adequate mechanical integrity and water stability [17]. To the best of our knowledge, a strategy that achieves structurally resilient polysaccharide foams using DES as the sole structuring agent and with gelation under ambient processing conditions has not yet been explored.

This study addresses these limitations by developing CMC-based foams where DES acts as the sole structuring agent. A key innovation is the implementation of a room temperature gelation process, which eliminates the need for energy-consuming thermal curing or external chemical crosslinkers, followed by freeze-drying to produce the final foams. This approach aims to yield materials that combine high swelling capacity, robust compressive properties, and structural stability in aqueous environments. The developed foams are characterized by scanning electron microscopy (SEM) to evaluate pore morphology and Fourier transform infrared spectroscopy (FTIR) to investigate molecular interactions. To evaluate mechanical behavior and structural durability, uniaxial compression tests are employed, including cyclic loading-unloading to assess recovery capacity. Furthermore, swelling behavior and structural integrity are evaluated under various aqueous conditions. As a proof-of-concept application, the ability of these CMC-based foams to function as drug delivery platforms is investigated using resveratrol (RSV) as a model compound. In vitro release studies are performed under different pH conditions to assess the stimuli-responsive behavior, accompanied by an analysis of the drug release kinetics. It should be noted that this study is exploratory and mainly focuses on the physicochemical and mechanical performance of the foams. In vitro and in vivo biological assays remain necessary to validate future biomedical viability.

## 2. Materials and Methods

### 2.1. Materials and Chemicals

To prepare the DES, reagents such as choline chloride 99% (ChCl, from Thermo Scientific, Shanghai, China), betaine 98% (BET, from Acros Organics, Geel, Belgium), citric acid monohydrate >98% (CA, from Sigma-Aldrich, Darmstadt, Germany), oxalic acid 99.5% (OA, from Labkem, Premià de Dalt, Barcelona, Spain), ethylene glycol 99% (EG, from Sigma-Aldrich, Darmstadt, Germany), and glycerol ≥99.5% (GLY, from Sigma-Aldrich, Darmstadt, Germany) were used. Sodium carboxymethyl cellulose (CMC, Finnfix 10, from Nouryon, Amsterdam, The Netherlands) was used for foam formation. Resveratrol >99% (RSV, from TCI Chemicals, Zwijndrecht, Belgium) was used as a model compound for the drug delivery system. For the release tests, phosphate-buffered saline tablet (PBS, pH 7.2–7.6, from Sigma-Aldrich, Darmstadt, Germany), hydrochloric acid 37.0% (from TCI Chemicals, Zwijndrecht, Belgium), and sodium hydroxide 98.5% (from Acros Organics, Geel, Belgium) were used. Milli-Q water was produced in-house using a Millipore purification system (Merck, Darmstadt, Germany), and all other solvents were of analytical grade and used as received without further purification.

### 2.2. Deep Eutectic Solvent (DES) Preparation

Each DES was prepared by mixing the HBA (ChCl or BET) with different HBD (CA, OA, EG, GLY), at defined molar ratios, as shown in Table 1. The mixtures were heated at 80 °C under continuous stirring (250 rpm) using a sealed container for 1 h until a homogeneous transparent liquid was formed. The resulting DES were stored under anhydrous conditions until further use.

### 2.3. CMC-Based Foam Preparation

**Figure 1:**
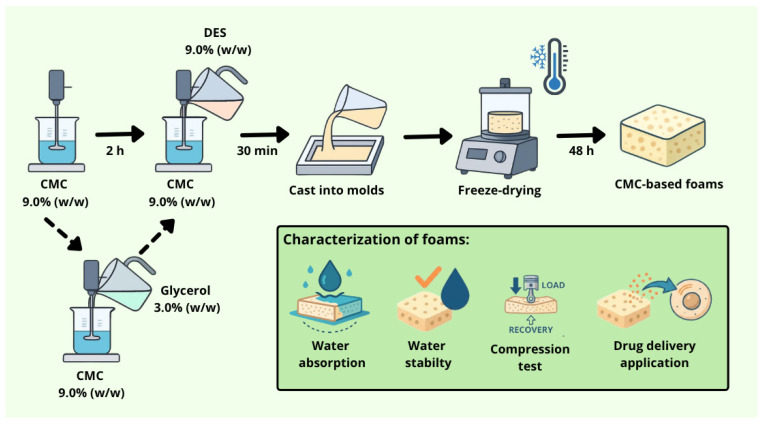
Schematic representation of the procedure adopted for the formation of CMC-based foams formulated with DES and/or glycerol, and some of the methods utilized for their characterization.

To prepare the CMC-based foams, a CMC-based hydrogel was first formed by dispersing CMC in water under mechanical stirring for 2 h at room temperature until complete solubilization. The polymer concentration was 9.0% (*w*/*w*). Next, the DES was added at 9.0% (*w*/*w*), and the mixture was stirred for 30 min at room temperature until a homogeneous system was obtained. The resulting mixture was cast into molds and freeze-dried for 48 h to obtain the foams. CMC-based foams containing glycerol were prepared following the same procedure, with glycerol (3.0% *w*/*w*) added during the initial dissolution step of CMC in water (Figure 1). A control experiment using separate components of DES instead of pre-formed DES was also performed by the addition of both HBA and HBD in the CMC-based hydrogel containing glycerol. Unless otherwise stated, all concentrations are expressed as weight percentage (% *w*/*w*) relative to the total mass of the hydrogel formulation at each preparation step.

### 2.4. CMC-Based Foams Loaded with Resveratrol

For the preparation of CMC-based foams loaded with RSV, CMC and glycerol were first dissolved in water (9.0% and 3.0% *w*/*w*, respectively) under mechanical stirring at room temperature. RSV (0.5% *w*/*w*) was then added directly to the formulation and mixed until a homogeneous dispersion was obtained. Subsequently, the corresponding DES (9.0% *w*/*w*) was added, and the system was stirred for 30 min to ensure homogeneity. The resulting mixture was cast into molds and freeze-dried for 48 h to obtain the final RSV-loaded foams, which were used for the in vitro release studies.

### 2.5. Foam Density and Porosity

The bulk density ($\rho_{bulk}$) of the CMC-based foams was calculated using the following formula (Equation (1)):

(1) $$\rho_{bulk}\;\left({g\;cm}^{-3}\right)=\left(W÷V\right),$$

where W is the weight of the foam and V is volume, which was calculated by measuring the height, length, and width of these foams [27].

Porosity (%) of the CMC-based foams was determined using a density-based method. Considering that shrinkage during drying was negligible, porosity was calculated using the following formula (Equation (2)):

(2) $$Porosity\left(\%\right)=\left[1-\left(\rho_{bulk}÷\rho_{solid}\right)\right]\times 100,$$

where $\rho_{solid}$ is the solid density, estimated from the composition of the hydrogel matrix [27].

### 2.6. Water Absorption

Water absorption ($W_{a}$) was evaluated by immersing the CMC-based foams with standardized dimensions (2 × 2 × 2 cm^3^) and previously weighed in Milli-Q^®^ water at room temperature (20–25 °C). At predetermined time intervals, samples were removed, gently blotted to remove excess surface water, and weighed. The water retention was calculated using the following formula (Equation (3)):

(3) $$W_{a}\left(\%\right)=\left[\left(W_{h}-W_{d}\right)÷W_{d}\right]\times 100,$$

where $W_{h}$ and $W_{d}$ are the weights of the hydrated and the freeze-dried foams, respectively. Long-term structural stability was assessed after 28 days of immersion by oven-drying the foams to constant weight and measuring their final dry mass. Mass retention was then calculated from the initial and final dry masses.

### 2.7. Foam Stability

The stability of the CMC-based foams was evaluated in water and in different pH solutions. Briefly, the CMC-based foams with standardized dimensions (2 × 2 × 2 cm^3^) were immersed in Milli-Q^®^ water, hydrochloric acid solution (pH 2.0), and sodium hydroxide solution (pH 13.0) at room temperature (20–25 °C). At predetermined time intervals over 28 days, the integrity of the foams was analyzed.

### 2.8. Compression Testing

To evaluate the mechanical behavior and structural durability of the CMC-based foams, uniaxial compression tests were performed on hydrated foams with standardized dimensions (2 × 2 × 2 cm^3^) using a Shimadzu EZ Test universal testing machine (Shimadzu, Vila Nova de Gaia, Portugal) equipped with a 200 N load cell. The experimental procedure was divided into two main categories: monotonic and cyclic testing. Monotonic compression tests were carried out at a strain rate of 0.03 mm s^−1^ to obtain stress–strain curves. The compression modulus (kPa), which measures the material’s stiffness, was calculated from the initial linear slope of these curves [27,28].

Subsequently, cyclic compression tests consisting of 10 consecutive cycles were performed to evaluate recovery, hysteresis, and performance degradation under repeated loading. These tests utilized a strain rate of 0.05 mm s^−1^, with samples compressed to a maximum strain ($\varepsilon_{max}$) of 50% of their original thickness, followed by an immediate unloading phase to allow recovery. Energy dissipation behavior was characterized by calculating the dissipated energy (*E_d_*), defined as the area within the hysteresis loop (the difference between the area under the loading and unloading curves). From these cycles, the loss coefficient (η) was determined to quantify the material’s ability to dissipate energy relative to the energy stored during the first compression (*E_s_*) using the following formula (Equation (4)) [29]:

(4) $$\eta =E_{d}÷E_{s},$$

Finally, the recovery (%) was assessed by comparing the maximum strain reached to the residual strain remaining after the load was fully removed using the following formula (Equation (5)):

(5) $$Recovery\left(\%\right)=\left[\left(\varepsilon_{max}-\varepsilon_{res}\right)÷\varepsilon_{max}\right)\times 100,$$

where $\varepsilon_{res}$ is the residual (minimum) strain after unloading.

### 2.9. Structure Characterization

#### 2.9.1. Scanning Electron Microscopy (SEM)

The surface morphology of the freeze-dried CMC-based foams was assessed by Scanning Electron Microscopy (SEM). Images were acquired using a Hitachi TM4000 plus microscope (Hitachi Scientific Instruments Ltd., Tokyo, Japan) operated at an accelerating voltage of 25 kV under low-vacuum conditions. The samples were mounted on aluminum stubs and analyzed without additional conductive coating. To determine the average pore size and pore-size distribution, at least 100 random pores from the SEM images were measured using ImageJ 1 software (National Institutes of Health, USA). The cumulative data was plotted into a frequency histogram overlaid with a Gaussian distribution curve using OriginPro 8.5 software (OriginLab Corp., Northampton, MA, USA).

#### 2.9.2. Fourier Transform Infrared–Attenuated Total Reflection (FTIR–ATR) Spectroscopy

FTIR-ATR spectra were recorded on a Perkin-Elmer FTIR System Spectrum BX spectrophotometer (Perkin-Elmer Inc., MA, USA) equipped with a single horizontal Golden Gate ATR cell. Spectra, with an average of 32 scans, were recorded in the range of 4000–600 cm^−1^ with a resolution of 4 cm^−1^. Baseline correction and spectral normalization were applied prior to analysis.

### 2.10. In Vitro Release of Resveratrol

The in vitro release of RSV from CMC-based foams was evaluated over 24 h in phosphate-buffered saline (PBS, pH 7.4), hydrochloric acid solution (pH 2.0), and sodium hydroxide solution (pH 13.0). Briefly, the RSV-loaded foams were immersed in 10 mL of release medium and maintained under constant stirring at ca. 37 °C. At predetermined time intervals (1, 2, 3, 4, 5, 6, 7, 8, 16, and 24 h), aliquots of 100 μL were withdrawn and the volume adjusted with fresh solutions. The amount of RSV released into the solution was quantified using a UV–Vis spectrophotometer (UV-2600, Shimadzu, Kyoto, Japan) at λ = 306 nm [30,31,32]. The drug released at each time was calculated as a cumulative release ($C_{cumulative}$) percentage using the following formula (Equation (6)):

(6) $$C_{cumulative}=C_{n}+\left[\left(2\times C_{n-1}\right)÷30\right],$$

where $C_{n}$ and $C_{n-1}$ are the RSV concentrations at time *n* and *n* − 1. The concentrations were estimated using a linear calibration curve ($R^{2}=0.997;\;y=88.049x+0.1587$; Figure S1) obtained by measuring the absorbance of RSV solutions (0.6–24.0 μg mL^−1^) at the same wavelength. Each experiment was performed in triplicate, and the average cumulative release was plotted as a function of time to evaluate the release profiles.

### 2.11. Drug Release Kinetics

The drug release data were fitted to the Korsmeyer–Peppas model [33] using the $M_{t}÷M_{\infty }=k_{m}t^{n}$ equation, where $M_{t}$ is the amount of drug released at time t (in hours), $M_{\infty }$ is the amount of drug released at infinite time, $k_{m}$ is the Korsmeyer–Peppas constant and n is the release exponent. In this case, only the values $M_{t}÷M_{\infty }$ < 60% are fitted. The coefficient of determination (R^2^) was used to determine the suitability of the model. The release exponent (n) confirmed the RSV transport mechanism for each CMC-based foam. As reported in the literature, n ≤ 0.5 indicates Fickian diffusion mechanism, 0.5 < n < 1.0 represents anomalous (non-Fickian) transport, and a value of n = 1.0 denotes Case-II transport. Values above 1.0 represent Super Case-II transport [33,34].

### 2.12. Statistical Analysis

All experiments were conducted in triplicate, and results are presented as means accompanied by standard deviation (SD). Statistical analyses were performed at a 95% confidence level using ANOVA to assess significant differences. A value of *p* < 0.05 was considered statistically significant, and Tukey’s multiple comparison test was applied for pairwise comparisons of means. All statistical analyses were performed using GraphPad Prism 10 software (GraphPad Software Inc., San Diego, CA, USA).

## 3. Results and Discussion

### 3.1. Screening of DES

Initially, six different types of DES were investigated for their ability to promote the formation of structurally stable CMC-based foams. Among the tested DES, ChCl and BET were selected as HBAs, whereas OA, CA, GLY, and EG were evaluated as HBDs. A key innovation of this work is the gelation process; while most literature relies on elevated energy-intensive high-temperature curing or external crosslinkers, the initial CMC-based hydrogels were successfully prepared at room temperature before undergoing freeze-drying for foam formation. This offers an alternative to traditional thermal-induced gelation and foam formation [23,29,35].

**Figure 2:**
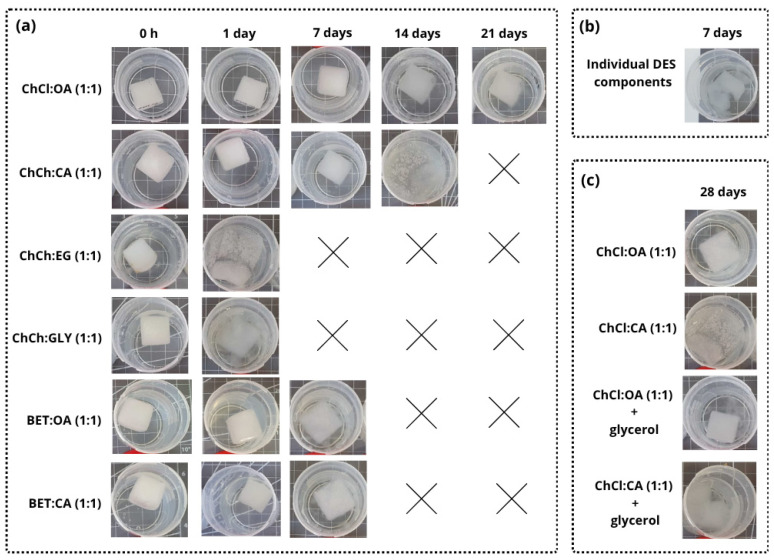
Assessment of the water stability of CMC-based foams formulated with: (**a**) the six different types of DES over 21 days; (**b**) separate components (ChCl and OA) over 7 days; (**c**) ChCl:OA (1:1) and ChCl:CA (1:1) with and without the addition of glycerol over 28 days.

Regarding water stability, a strong dependence of foam properties on the chemical nature of the DES components was observed. For instance, DES based on carboxylic acids, namely ChCl:CA (1:1) and ChCl:OA (1:1), produced foams with superior water stability (Figure 2a). This behavior can be attributed to the ability of carboxylic acids to form strong hydrogen bonds with the hydroxyl and carboxylate groups of CMC, promoting stable supramolecular structuring [36,37,38].

In contrast, DES containing polyols as HBD, namely ChCl:GLY (1:1) and ChCl:EG (1:1), resulted in foams that rapidly dissolved in water (Figure 2a). Although GLY and EG can participate in hydrogen bonding, their interactions with CMC are weaker, leading to less stable network formation. This observation is consistent with previous reports describing that polyol-based DES mainly promotes swelling and plasticization without causing significant chemical modification in the polymer backbone [39]. As a result, polyol-based DESs probably behave only as plasticizers, rather than structuring agents, resulting in foams with insufficient structural integrity, especially upon immersion in water.

The nature of the HBA also influences the properties of the resulting foams. DES based on betaine (BET:CA and BET:OA) produced fragile materials with poor water stability (Figure 2a). This behavior may be related to the distinct structural and interaction characteristics of BET compared to ChCl. While ChCl has charges that are physically located in two independent ions (choline and chloride), betaine is a zwitterionic compound with internally compensated charges. In addition, betaine has a carboxylic group while ChCl has a hydroxyl group. Thus, their charge interactions are expected to differ. ChCl may interact more strongly with CMC, reducing electrostatic repulsion and increasing intermolecular interactions [40].

To verify whether the mechanical behavior and structural stability of the CMC-based foams could be further improved, the incorporation of glycerol alongside the carboxylic acid-based DES (ChCl:OA and ChCl:CA) was investigated. The addition of glycerol to CMC-based materials has been reported to enhance flexibility and improve material strength [41]. Herein, the results confirm this trend by showing that the addition of glycerol increases structural integrity and, as a result, the CMC-based foams were stable for more than 28 days, especially the one produced using ChCl:OA (1:1) (Figure 2c).

Moreover, to better understand the role of the carboxylic acid-based DES in the formation of stable foams, a control experiment using the addition of the separate DES components (ChCl and OA) instead of the pre-formed DES was performed. As can be seen in Figure 2b, this type of foam led to the formation of less stable materials when compared to the ones prepared with DES, resulting in their total solubilization after 7 days.

Based on these screening results, ChCl:OA (1:1) was identified as the most effective DES for producing stable CMC-based foams, particularly when combined with glycerol. This formulation was therefore selected for further foam preparation and mechanical and morphological characterization, together with control samples prepared by adding separate DES components (ChCl and OA) and glycerol, rather than the pre-formed DES.

### 3.2. Morphological Analysis

**Figure 3:**
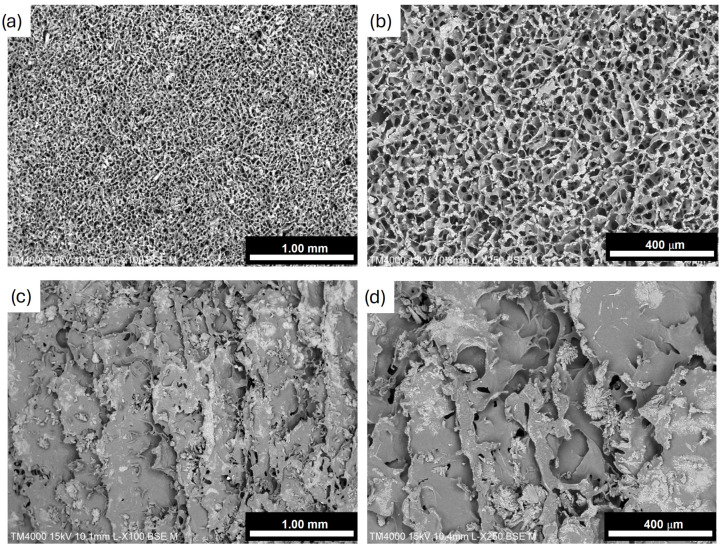
SEM images of: (**a**) CMC-based foams prepared with DES and glycerol (100× magnification); (**b**) CMC-based foams prepared with DES and glycerol (250× magnification); (**c**) CMC-based foams prepared with separate components and glycerol (100× magnification); (**d**) CMC-based foams prepared with separate components and glycerol (250× magnification).

SEM imaging was used to qualitatively assess morphology and to quantitatively assess pore size. The SEM images of the CMC-based foams prepared with the select DES (ChCl:OA 1:1) containing glycerol (Figure 3a,b) reveal a highly porous and well-defined network with a relatively uniform distribution of pore sizes (32.2 ± 13.3 µm) (Figure S2). This structural uniformity is supported by the physical characterization of the foams, which exhibit a bulk density ($\rho_{bulk}$) of 0.2024 g cm^−3^ and a high calculated porosity of 85.01%. This architecture is consistent with other cellulose-based foams obtained from cellulose derivatives by freeze-drying, which normally show low density (up to 0.35 g cm^−3^), high porosity (80~99.8%), and macropores (pore size ≥50 nm) [42,43].

In contrast, the foams prepared with separate DES components and glycerol (Figure 3c,d) exhibit a much more irregular and collapsed morphology, characterized by thicker and denser wall structures, as well as large and non-uniform voids. The lack of structural uniformity in these foams suggests that without the pre-established hydrogen bonding network of DES, the individual components probably undergo phase separation during the freeze-drying process. Based on that, the pre-formed DES could facilitate a more homogeneous dispersion of the CMC chains within the aqueous medium, preventing the agglomeration of components during the freeze-drying process. This results in a more interconnected and organized porous framework, as evidenced by the high percentage of void space (85.01%) maintained in the final foam.

Similar results were observed by Iyer et al. with bacterial cellulose-based foams prepared in the presence of ChCl:CA (1:1), where without the DES treatment, the cellulose agglomerates into fiber-rich and water-rich phases, leading to clusters of fibers and large pore sizes. With the DES treatment, the fibers are less agglomerated and therefore create more uniform pore sizes (117 ± 73 μm). Our results follow a similar trend, in which the inclusion of DES leads to a more refined micro-architecture with smaller and more controlled pore dimensions [29].

### 3.3. Water Absorption Performance

The water absorption capacity of the CMC-based foams prepared with DES and glycerol was evaluated to determine their ability to hydrate and maintain structural integrity in an aqueous environment. The foams exhibited a rapid swelling of 288.24 ± 3.02% within the first 1 h of immersion. This fast hydration kinetics is directly linked to the highly porous and interconnected micro-channel architecture observed in the SEM analysis (Section 3.2), which allows for efficient water transport via capillary action throughout the scaffold.

Interestingly, the swelling degree remained constant, 289.71 ± 5.63% and 300.00 ± 4.26% after 48 and 96 h, respectively. This indicates that the foams reach a state of equilibrium very quickly and, more importantly, that the physical network formation established by the DES and glycerol is robust enough to prevent the polymer network from dissolving over extended periods in water. This behavior contrasts with the control samples prepared with separate DES components and glycerol (242.24 ± 2.75% after 1 h).

To quantitatively support the long-term water stability, mass retention measurements were performed on the optimized CMC-based foams. The data revealed that the foams successfully retained 88.4 ± 1.8% of their initial dry mass after 28 days of immersion. This high mass retention confirms the strong interactions between the pre-formed DES and CMC.

### 3.4. Mechanical Behavior

**Figure 4:**
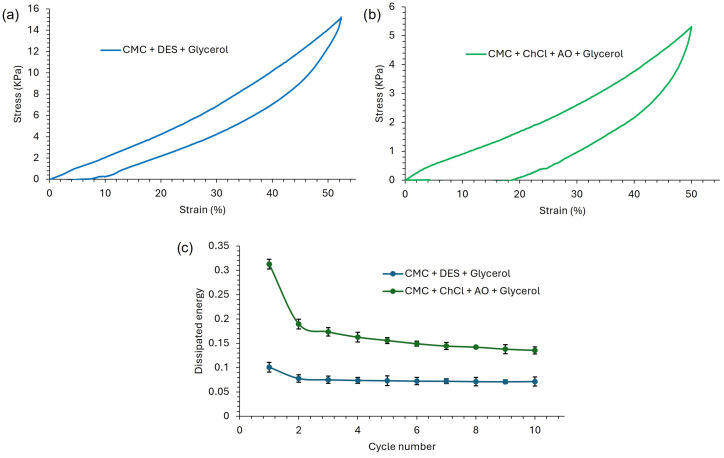
Mechanical tests: (**a**) stress–strain curve first cycle of CMC-based foams prepared with DES and glycerol; (**b**) stress–strain curve first cycle of CMC-based foams prepared with separate DES components and glycerol; (**c**) dissipated energy throughout 10 cyclic compression testing.

Given their highly porous and layered structure, the mechanical behavior of the hydrated CMC-based foams was evaluated using uniaxial and cyclic compression tests. As shown in the representative stress–strain curves (Figure 4a,b), the CMC-based foams prepared with ChCl:OA (1:1) and glycerol exhibited significantly higher compressive strength, reaching a peak stress of 15.23 ± 0.55 kPa at 50% strain, nearly three times higher than the foams prepared with separate components (ChCl and OA) instead of pre-formed DES (5.32 ± 0.29 kPa). This correlates with the compression modulus (Table 2), which was notably higher for the foams made with DES (19.48 ± 2.01 kPa) than for the separate components (7.46 ± 1.19 kPa).

These differences in mechanical behavior could be explained by comparing their morphological characteristics (Section 3.2). For instance, the uniform structure of the CMC-based foams prepared with DES allows for a symmetric stress distribution, leading to a significantly higher compression modulus (19.48 ± 2.01 kPa) and superior recoverability (91.92 ± 4.57%). Conversely, the disordered and dense regions seen in the CMC-based foams prepared with the separate components suggest that, without the stabilizing hydrogen-bonding network of the DES, the individual ChCl and OA molecules likely induce phase separation or localized CMC clustering. This structural heterogeneity creates weak points that lead to the lower compressive strength (5.3 kPa) and the high degree of permanent deformation observed in the cyclic tests.

Analysis of the energy dissipation profiles (Figure 4c) shows that the CMC-based foams experienced a sharp decrease in dissipated energy ($E_{d}$) after the initial cycle before reaching a steady-state response. However, the CMC-based foams prepared with pre-formed DES and glycerol demonstrated a remarkably low loss coefficient (3.35 ± 0.01%) in the first cycle compared to the separate components (26.99 ± 1.51%), indicating that these foams store energy ($E_{s}$) much more efficiently than they dissipate. Once again, the morphological characteristics observed in the SEM images, especially in the higher magnification images, allow us to understand the energy dissipation profiles. The CMC-based foams prepared with DES (Figure 3b) show walls consisting of cohesive, sheet-like assemblies, which contribute to the material’s ability to store energy (*E_s_*) efficiently with minimal dissipation. In the foams made with separate components (Figure 3d), morphology appears fractured and less continuous, which explains the high loss coefficient (26.99 ± 1.51%) as energy is likely dissipated through friction between non-interconnected structural fragments.

Furthermore, the foams made with DES and glycerol maintained an exceptional recovery rate of 93.29 ± 3.12% over 10 cycles, while the foams made with separate components showed significant permanent deformation, with recovery dropping to 56.92 ± 2.01% (Table 2). This stability under repeated loading confirms that the synergy within the DES is essential for enhancing the foam’s elasticity and fatigue resistance, preventing the structural breakdown observed when the components are added separately.

Ultimately, the use of a pre-formed DES is critical for achieving a refined and stable porous morphology, which translates directly into enhanced mechanical stability and recovery, parameters required for advanced foam applications, compared to the simple addition of the DES’s separate components.

### 3.5. FTIR-ATR Analysis

**Figure 5:**
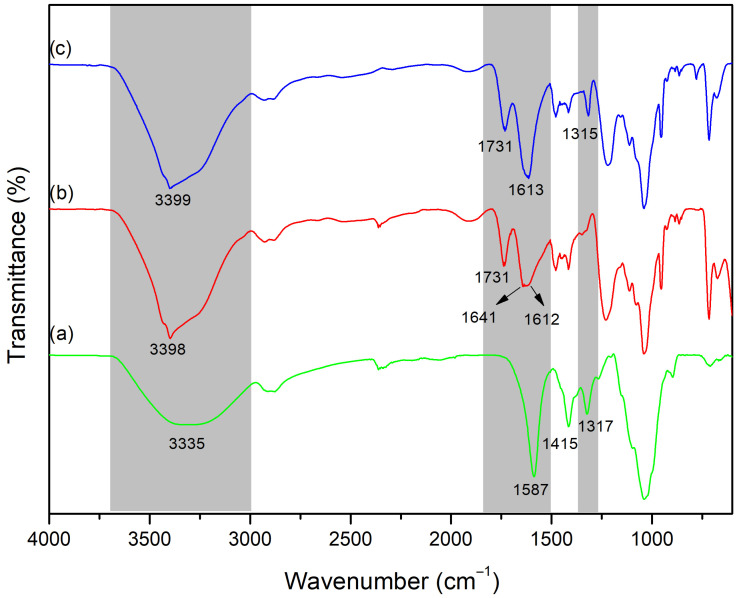
FTIR-ATR spectra of (a) pure CMC; (b) CMC-based foams prepared with DES and glycerol; (c) CMC-based foams prepared with separate components and glycerol.

To investigate possible molecular changes in CMC due to the presence of DES that could explain the superior behavior observed in the water stability, mechanical tests, and SEM images, FTIR-ATR was performed. As shown in Figure 5, both CMC-based foams (foams prepared with pre-formed DES and foams prepared with separate components) have broad O-H stretching vibrations (3000–3800 cm^−1^) and C=O stretching vibrations (1600–1800 cm^−1^), indicating the presence of multiple interaction environments. A shift in the characteristic O-H band from the initial CMC of 3335 cm^−1^ (Figure 5a) to approximately 3398 cm^−1^ in the foams suggests an increased density of hydrogen-bonded hydroxyl groups resulting from the interaction between the CMC backbone and the oxalic acid (OA), a fact that has already been reported in the literature [44].

The most critical evidence suggesting intermolecular interactions between CMC and DES is found in the 1600 cm^−1^ region. In the foams prepared with pre-formed DES and glycerol (Figure 5b), a significant broadening and splitting occur between 1612–1641 cm^−1^, which could suggest coordinated interactions, probably hydrogen bonding and electrostatic interactions, between the DES and the CMC carboxylate groups [36,37]. In contrast, the foams prepared with separate components and glycerol (Figure 5c) display a sharp, single peak at 1613 cm^−1^ and a distinct band at 1315 cm^−1^ (O–H bending vibration of CMC). The presence of this 1315 cm^−1^ band suggests that when components are added separately, the CMC retains more free functional groups. In this case, each component probably interacts independently with CMC, leading to the weaker bonding and structural instability observed in mechanical analysis and water stability. Therefore, preliminary spectra suggest potential interactions between DES components and CMC carboxylate groups.

### 3.6. Foam Stability in Different pH

**Figure 6:**
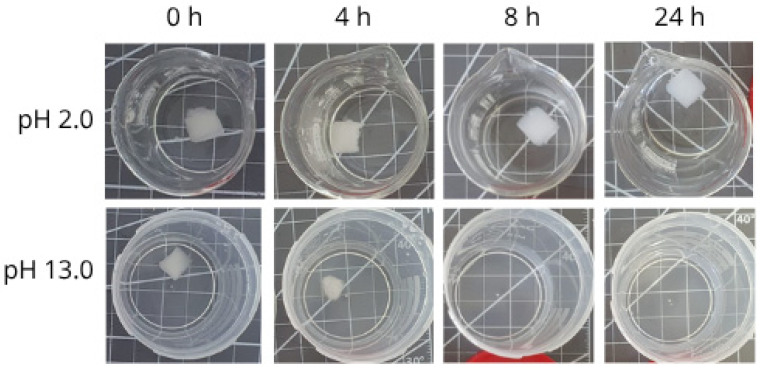
Assessment of the stability of CMC-based foams prepared with ChCl:OA (1:1) and glycerol at pH 2.0 and 13.0 over 24 h.

The stability of CMC-based foams prepared with ChCl:OA (1:1) and glycerol was evaluated under acidic (pH 2.0) and alkaline (pH 13.0) conditions over 24 h (Figure 6). The results reveal a strong dependence of foam stability on pH, highlighting the sensitivity of the system to protonation-deprotonation equilibria and intermolecular interactions [45]. At pH 2.0, the foams demonstrate exceptional structural integrity, remaining virtually unchanged throughout the experiment. This is primarily attributed to the protonation of the carboxyl groups (-COOH) in the CMC backbone, which significantly reduces electrostatic repulsion between polymer chains, leading to a more cohesive and mechanically resistant foam [45].

In contrast, at pH 13.0, the foams exhibited rapid destabilization, with significant structural collapse occurring within the first hours, and underwent complete dissolution after 24 h. Under highly alkaline conditions, the carboxylic groups of CMC are fully deprotonated (-COO^−^), resulting in a high density of negative charges along the polymer backbone [45]. This leads to intense electrostatic repulsion between the negatively charged polymer chains, effectively breaking the physical network formation. Moreover, this result could also be indicative that covalent interactions between CMC and the oxalic acid component from the DES exist and are broken down in an alkaline environment.

This pH-responsive behavior suggests that CMC-based foams prepared with ChCl:OA (1:1) and glycerol remain stable under acidic conditions but readily destabilize and dissolve in a more alkaline medium. This makes them promising candidates for pH-triggered delivery systems, where the structure is preserved in acidic environments and controlled disassembly at higher pH enables the release of active compounds.

Furthermore, possible leaching of DES components should be considered during CMC-based foam dissolution under different pH conditions. For that, a theoretical worst-case scenario was established assuming the total and instantaneous leaching of DES components from a standard foam unit sample (2 × 2 × 2 cm^3^). Considering that a typical hydrogel formulation contains 9.0 wt% of DES and that the average dry foam has ca. 1.6 g, the maximum possible chemical leaching per single foam is 144.0 mg of total DES (72.6 mg of ChCl and 71.4 mg of OA). According to literature, in vitro toxicity data on cell lines indicate that ChCl:OA (1:1) possesses moderate cytotoxicity, with EC_50_ values of 1.64 mM (218.7 mg L^−1^) and 4.19 mM (558.98 mg L^−1^) for fish (CCO) and human (MCF-7) cell lines, respectively [46]. Given that the total DES loaded in a single foam would be widely diluted under standard physiological conditions, the final exposure concentrations should remain below these literature thresholds.

### 3.7. In Vitro Release Behavior of Resveratrol

To assess whether CMC-based foams made with the optimized DES can function as drug delivery systems, RSV was selected as a model drug and incorporated into foams prepared with ChCl:OA (1:1) containing glycerol. For this purpose, 0.5% (*w*/*w*) of RSV was added to the CMC-based hydrogel before freeze-drying. RSV was chosen as the drug model compound due to its poor water solubility, low stability, and limited bioavailability. Additionally, RSV is widely recognized for its therapeutic potential in the treatment of gastrointestinal diseases, and several studies have focused on enhancing its stability under acidic gastric conditions (pH 1.0–2.0) while enabling controlled release in the intestinal environment (pH 5.5–7.4) [47,48]. This is consistent with our previous observations regarding the stability behavior of CMC-based foams in different pH conditions (Section 3.6).

**Figure 7:**
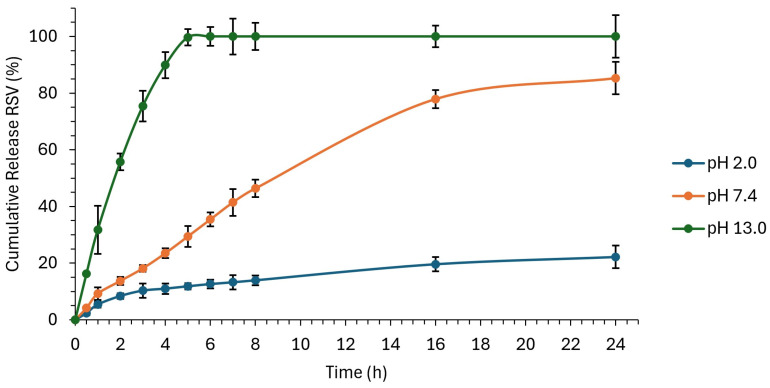
Cumulative release profile of resveratrol from CMC-based foams over time (hours) in HCl solution (pH 2.0), PBS solution (pH 7.4), and NaOH solution (pH 13.0).

Therefore, to mimic gastrointestinal conditions, the in vitro release of RSV from CMC-based foams was evaluated in three different pH conditions. Hydrochloric acid solution (pH 2.0) was used to mimic the gastric environment. PBS (pH 7.4) was chosen to understand the drug delivery profile under simulated intestinal conditions. Finally, sodium hydroxide solution (pH 13.0) was used for comparative purposes (not a physiological pH). Moreover, the in vitro release profiles were monitored for 24 h, a timeframe chosen to closely mimic the typical physiological transit time of oral delivery systems through the human gastrointestinal tract. As illustrated in Figure 7, an increase in pH corresponds to an enhanced release rate of RSV. For instance, at pH 13.0, cumulative drug release reached 55.73 ± 2.95% in 2 h and 100% by 5 h. At pH 7.4 and 2.0, releases after 2 h were only 13.70 ± 1.42% and 8.41 ± 1.03%, with maximum RSV cumulative release after 24 h at 85.30 ± 5.75% and 22.18 ± 3.99%, respectively.

To better understand how RSV is released from CMC-based foams, the experimental data were analyzed using the Korsmeyer–Peppas kinetic model (Figure S3). Based on this model, we obtained a release exponent of n=0.42 (R^2^ = 0.984) for pH 2.0, n=0.79 (R^2^ = 0.984) for pH 7.4, and n=0.76 (R^2^ = 0.997) for pH 13.0. The regression coefficients are in the permissible range for all analyses (R^2^ ≥ 0.95), indicating the model’s suitability. The release exponent of RSV in HCl solution (pH 2.0) is representative of a Fickian transport mechanism (n ≤ 0.5), suggesting that the release of RSV from the CMC-based foams in an acidic environment is essentially governed by the simple diffusion of the drug molecules through the foam [34].

In contrast, the higher values of the release exponent obtained for the RSV release in PBS (pH 7.4) and NaOH solutions (pH 13.0) are indicative of anomalous (non-Fickian) transport (0.5 < n < 1.0) [34]. This suggests that in a more alkaline pH, RSV release diverges from classical diffusion patterns and involves additional processes, such as swelling and partial structural disintegration of the CMC-based foams, which contributes to a more intricate release profile. Importantly, this kinetic transition is consistent with the macroscopic observation of foam disappearance at alkaline pH (Figure 7), confirming that drug release is not only diffusion-controlled but also governed by structural breakdown of the CMC-based foams. Moreover, the difference in behavior observed in this study is in accordance with the results previously reported for CMC-based hydrogels, where a Fickian behavior was observed at pH 1.4 and non-Fickian behavior at pH 11.7 [49].

Therefore, the developed CMC-based foams show promise as pH-responsive drug delivery platforms, particularly for oral drug administration, where protecting active compounds in acidic gastric conditions (pH 1.0–2.0) and enabling controlled release in intestinal environments (pH 5.5–7.4) are essential [50,51]. Their stability at low pH supports their role as protective carriers that limit premature drug loss, whereas the higher release at pH 7.4 suggests suitability for intestinal delivery. Although pH 13.0 is not physiologically relevant, it provides a useful stress test that highlights the system’s structural limits, showing ionization-driven collapse of the foam network. Together, these results indicate a pH-dependent structural switch that governs drug transport, which is particularly relevant for RSV given its limited stability and bioavailability in the gastrointestinal tract [47,48].

## 4. Conclusions

This study successfully demonstrated a simplified approach for the development of CMC-based foams using DES as the sole multifunctional structuring agent. By enabling hydrogel formation at room temperature and avoiding the use of external chemical crosslinkers, the proposed approach contributes to more sustainable processing of polysaccharide-based materials. Among the systems investigated, the combination of ChCl:OA (1:1) with glycerol yielded foams with the most favorable balance of structural integrity, water stability, and mechanical performance. Moreover, the results indicate that the use of pre-formed DES plays a critical role in defining the final material properties. Compared to foams prepared using separate DES components (ChCl and OA), DES-based formulations produced more homogeneous and interconnected porous structures, which translated into improved compressive strength and a high recovery rate of 93.29 ± 3.1% over 10 compression cycles. FTIR-ATR analysis suggests that these effects may arise from enhanced intermolecular interactions, particularly hydrogen bonding between CMC and DES, consistent with the preliminary spectral evidence of potential interactions within the CMC-DES network.

The developed CMC-based foams exhibited high water uptake (288.24 ± 3.02% after 1 h) and high stability in aqueous environments for at least 28 days, as well as clear pH-dependent behavior. Resveratrol release studies showed limited release under acidic conditions (22.18 ± 3.99%) and higher release at pH 7.4 (85.30 ± 5.75%) after 24 h, with kinetic analysis indicating a transition from diffusion-controlled to anomalous transport mechanisms. These results highlight CMC-based foams prepared with DES as tunable and stimuli-responsive porous matrices with potential applicability as pH-dependent delivery platforms.

However, because this work mainly focuses on sustainable processing and exploratory physicochemical characterization, the current findings serve as a fundamental engineering prototype. To successfully transition these CMC-based foams into true biomedical contexts, further studies addressing technical scale-up hurdles, such as freeze-drying batch consistency, terminal sterilization impacts, and product shelf-life, are required. Furthermore, extensive biological validation, including in vitro cytocompatibility assays, hemocompatibility testing, and long-term degradation kinetics under enzyme-containing simulated physiological fluids, remains crucial to fully assess their therapeutic safety and structural applicability. While the present pH-responsiveness study establishes the purely chemical dissolution behavior of the matrices, future work should also encompass long-term profiles in enzyme-containing simulated gastrointestinal fluids (SGF/SIF). Overall, this study highlights the ability of DES to modulate the structure and performance of CMC-based foams, offering a promising, green alternative to conventional chemical crosslinking strategies.

## Figures and Tables

**Table 1:** DES composition (HBA and HBD) and their molar ratio.

DES	HBA	HBD	Molar Ratio HBA:HBD
1	ChCl	CA	1:1
2	ChCl	OA	1:1
3	ChCl	EG	1:1
4	ChCl	GLY	1:1
5	BET	CA	1:1
6	BET	OA	1:1

**Table 2:** Values of recovery (%), compression modulus (kPa), dissipated energy (kJ m^−3^), stored energy (kJ m^−3^), and loss coefficient (%) of CMC-based foams prepared with DES and separate components in the first and tenth cyclic compression test.

CMC-Based Foam Prepared With	Cycle	Recovery (%) *	Compression Modulus (kPa) *	Dissipated Energy (kJ m^−3^) *	Stored Energy (kJ m^−3^) *	Loss Coefficient (%) *
DES **	1	91.92 ± 4.57	19.48 ± 2.01	0.10 ± 0.00	3.01 ± 0.01	3.35 ± 0.01
DES **	10	93.29 ± 3.12	12.62 ± 1.10	0.07 ± 0.00	2.42 ± 0.01	2.93 ± 0.01
Separate components **	1	62.58 ± 2.35	7.46 ± 1.19	0.31 ± 0.00	1.15 ± 0.05	26.99 ± 1.51
Separate components **	10	56.92 ± 2.01	3.07 ± 0.51	0.13 ± 0.00	0.65 ± 0.02	20.58 ± 1.12

* Data are presented as mean ±standard deviation (SD). ** Containing glycerol.

## Data Availability

The original contributions presented in this study are included in the article/Supplementary Material. Further inquiries can be directed to the corresponding author.

## Supplementary Materials

The following supporting information can be downloaded at: https://www.mdpi.com/article/10.3390/jfb17070337/s1. Figure S1. Calibration curve of resveratrol. Figure S2. (a) SEM images of CMC-based foams prepared with DES and glycerol (250× magnification) with pore sizes; (b) Pore size distribution histogram. Figure S3. Korsmeyer–Peppas kinetic model for the resveratrol release from CMC-based foams at (a) pH 2.0; (b) pH 7.4; (c) pH 13.0. Figure S4. Water absorption (%) of CMC-based foams prepared with DES and glycerol, and CMC-based foams prepared with separate components and glycerol after 1 h, 48 h, and 96 h.

## Abbreviations

The following abbreviations are used in this manuscript:

BET	Betaine
CA	Citric acid
ChCl	Choline chloride
CMC	Carboxymethyl cellulose
DES	Deep eutectic solvent
EG	Ethylene glycol
FTIR	Fourier transform infrared spectroscopy
GLY	Glycerol
HBA	Hydrogen bond acceptor
HBD	Hydrogen bond donor
OA	Oxalic acid
PBS	Phosphate-buffered saline
RSV	Resveratrol
SD	Standard deviation
SEM	Scanning electron microscopy
