# Supplementary material for: Sustainable Carboxymethyl Cellulose-Based Foams via Deep Eutectic Solvent Processing for pH-Responsive Drug Delivery
Source: J Funct Biomater. 2026 Jul 12;17(7):337. doi: 10.3390/jfb17070337 (PMC13412967; doi:10.3390/jfb17070337)
Supplement: Supplementary file 1 [file jfb-17-00337-s001.zip › jfb-4390900-supplementary.pdf]

Supplementary Materials

Sustainable Carboxymethyl Cellulose-Based Foams via Deep Eutectic Solvent Processing for pH-Responsive Drug Delivery

Bruno B. Ravanello [1], Filipe Silva de Matos [1], Bruna Ramos Navalhas [1], Luís Pereira [1,2] and Nalin Seixas [3,*]

[1] AlmaScience Colab, Madan Parque, Rua dos Inventores, 2825-182 Caparica, Portugal; bruno.ravanello@almascience.pt (B.B.R.); filipe.matos@almascience.pt (F.S.d.M.); bruna.navalhas@almascience.pt (B.R.N.); lmnp@fct.unl.pt (L.P.)

[2] CENIMAT|i3N, Department of Materials Science, School of Science and Technology, NOVA University Lis-bon and CEMOP/UNINOVA, Campus da Caparica, 2829-516 Caparica, Portugal

[3] CCICECO—Aveiro Institute of Materials, Department of Chemistry, University of Aveiro, 3810-193 Aveiro, Portugal

* Correspondence: nalinseixas@ua.pt

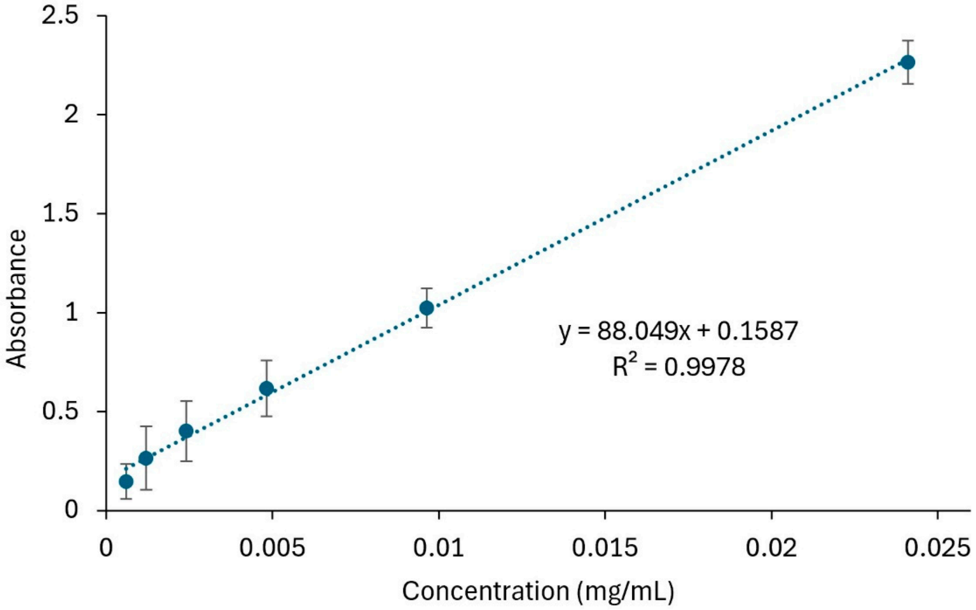

Figure S1. Calibration curve of resveratrol.

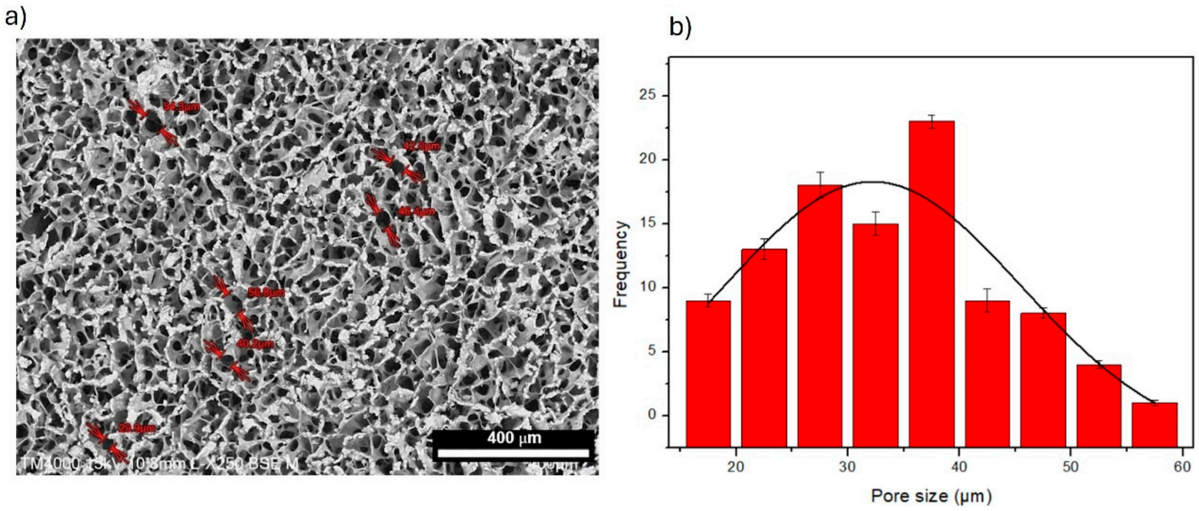

Figure S2. (a) SEM images of CMC-based foams prepared with DES and glycerol (250X magnification) with pore sizes; (b) Pore size distribution histogram.

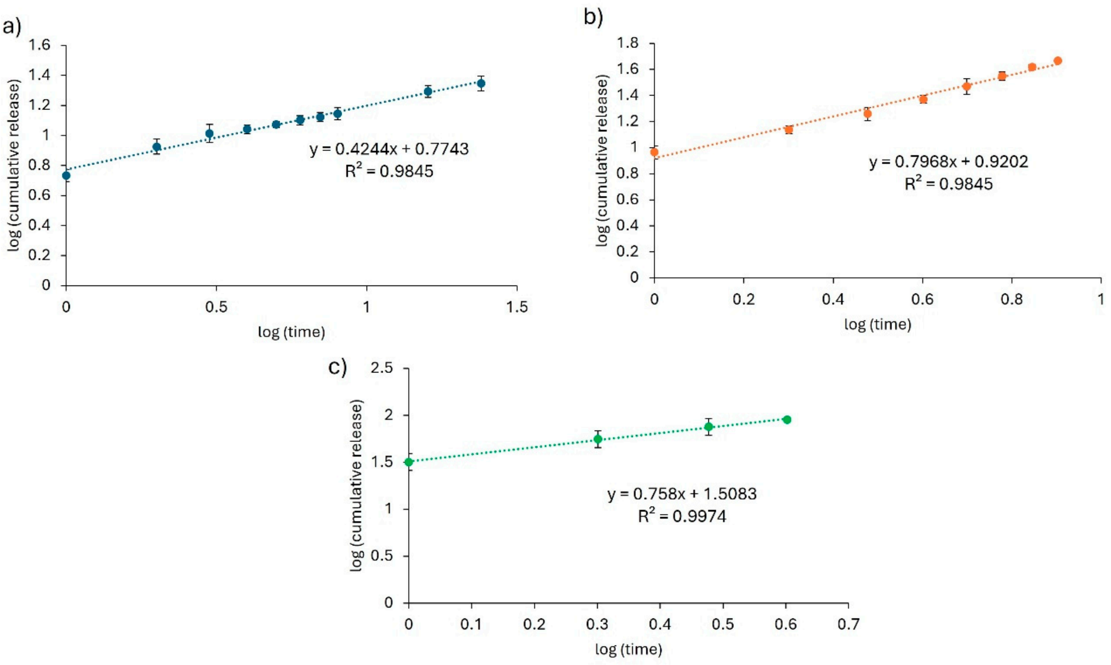

Figure S3. Korsmeyer–Peppas kinetic model for the resveratrol release from CMC-based foams at **(a)** pH 2.0; **(b)** pH 7.4; **(c)** pH 13.0.

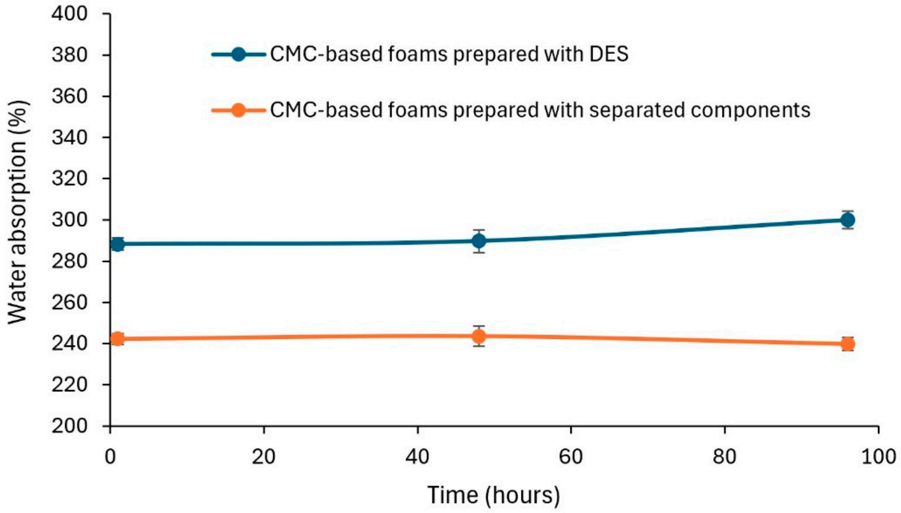

Figure S4. Water absorption (%) of CMC-based foams prepared with DES and glycerol, and CMC-based foams prepared with separate components and glycerol after 1 h, 48 h, and 96 h.
